# Risk factors of severe hepatotoxicity among HIV-1 infected individuals initiated on highly active antiretroviral therapy in the Northwest Region of Cameroon

**DOI:** 10.1186/s12876-022-02305-x

**Published:** 2022-06-03

**Authors:** Lem Edith Abongwa, Anthony Kebira Nyamache, Fokunang Charles, Judith Torimiro, Nshom Emmanuel, Irénée Domkam, Mbu Eyongetah, Beriyuy Jude, Fung Holgar Mua, Sama Bella, Tankou Colman Tamboh, Erna Charlene Moungang, Victorine Ngum, Paul Okemo

**Affiliations:** 1 Department of Biological Sciences, Faculty of Science, University of Bamenda, BP 39, Bamenda, North West Region Cameroon; 2 Department of Microbiology, School of Pure and Applied Sciences, Kenyatta University, Nairobi, Kenya; 3 Chantal Biya International Center for Research on the Prevention and Management of HIV/AIDS (CIRCB), Yaoundé, Cameroon; 4 Faculty of Medicine and Biomedical Sciences, University of Yaoundé I, Yaoundé, Cameroon; 5 Baptist Convention Health Board, Mbingo Baptist Hospital, Bamenda, North West Region Cameroon; 6 Bamenda Regional Hospital, Bamenda, North West Region Cameroon; 7 Santa district health centre, Santa, North West Region Cameroon; 8 Ndop district health centre, Ndop, North West Region Cameroon; 9 Bali District Health Centre, Bali, North West Region Cameroon; 10 Bafut District Hospitals, Bafut, North West Region Cameroon

**Keywords:** Risk factors, Liver enzymes, HAART, HIV-1, Hepatotoxicity

## Abstract

**Background:**

Hepatotoxicity due to highly active antiretroviral therapy (HAART) has gained prominent attention since it can be affected by many factors. The aim of this study was to determine the prevalence of hepatotoxicity and related risk factors of severe hepatotoxicity following HAART initiation.

**Methods:**

A total of 100 drug-naive patients aged between 18 and 61 years were recruited. They were put on Tenofovir/Lamivudine/Efavirenz [TDF/3TC/EFV] (64), Zidovudine/ Lamivudine/Efavirenz [AZT/3TC/EFV] (22), and Zidovudine/Lamivudine/Nevirapine AZT/3TC/NVP (14) and monitored for 6months and blood samples drawn.Alanine aminotransferases (ALT), aspartate aminotransferases (AST), and alkaline phosphatase (ALP) wereanalyzed by enzymatic methods and used to classify levels of hepatotoxicity.

**Results:**

A total of 37(37%) and 49(49%) patients presented with hepatotoxicity while 15% and 28% had severe hepatotoxicity at 4 and 24 weeks respectively. Serum levels of all enzymes increased significantly (*p* = 0.001) with increased treatment duration. Univariate analysis revealed that the risk factor of developing severe hepatotoxicity was significantly greater in patients < 30years (*p* = 0.02), males(*p* = 0.04), low BMI (*p* = 0.02), low monthly income (*p* = 0.01) earners, and patients on AZT + 3TC + NVP regimen (*p* = 0.01). While multivariate analysis at p < 0.09 showed that age 30–40 years, low BMI, low monthly income, and the use of AZT + 3TC + NVP regimen were independent risk factors.

**Conclusions:**

Low BMI, age group of 30–40years, low monthly income, and the use of AZT + 3TC + NVP regimen identified as risk factors for the development of severe hepatotoxicity should be considered as an important strategy by clinicians in preventing the hepatotoxicity.

## Introduction

1–3]. However, these HAART have also been reported to induce severe or life-threatening cases of adverse effects such as hepatotoxicity which have been the most important limiting factors to the successful use of HAART [3]. In the era of HAART, hepatotoxicity prevalence range of 1–54.0% and has been attributed to the discontinuation of HAART which has increased the morbidity and mortality rates among patients infected with HIV [2–7].

Several studies have been conducted to determine the risk factors of hepatotoxicity in patients with HIV. These factors include age, gender, type of HAART, viral load, hypertryglyceridemia, hyperglycemia, history of tuberculosis therapy and hepatitis B and Hepatitis C co-infection, alcohol abuse, higher baseline ALT or AST [3, 5, 7–10]. However, these findings cannot be universally applicable based on diverse regions, cultures, and specific human adaptations making it impossible to extrapolate the information from one population to another. Thus this study aimed to determine the prevalence of hepatotoxicity and related risk factors of severe hepatotoxicity among patients infected with HIV on HAART.

## Methods

### Study design and population

This was a hospital-based longitudinal analytical study conducted at the HIV Outpatient Clinic in Ndop, Santa, Bali, and Bafut District Hospitals and the Regional hospital of Bamenda of the NWR of Cameroon from February 2016 to November 2016. The sampled sites better represent the different backgrounds of individuals from the entire NWR of Cameroon. Bafut, Santa, Bali, and Ndop are in rural areas while Bamenda is from urban settings. The National Ethics Committee of Cameroon approved the study protocols with ethical approval number No 2016/01/689/CE/CNERSH/SP, and all patients gave their written consent before enrolment. The eligibility criteria included a confirmed HIV-1 infection, HAART naïve, negative for viral hepatitis B or C, not on tuberculosis treatment, and willingness to be followed up for at least 24 weeks.

Data were obtained with the aid of a pre-tested questionnaire and with the consultation of the patient’s record. These include information on; age, sex, weight, level of education, monthly income, history of alcohol and cigarette use, type of HAART, concomitant use of other drugs within 4 weeks before enrolment, WHO clinical staging, and the year the patient was first diagnosed of HIV. Patients routinely visited the HIV clinic every 4 weeks and were either on Tenofovir (TDF) + Lamivudine (3TC) + Efavirenz (EFV) or Zidovudine (AZT) + Lamivudine + Nevirapine (NVP) or AZT + 3TC + EFV according to Cameroonian HIV-1 first-line treatment guidelines. On each of these visits, clinical evaluation and assessment for adherence to HAART using self-report and pill counts were performed.

### Laboratory evaluations

About five ml of venous blood was collected from each subject and processed to obtain serum. Measurement of Alanine aminotransferase (ALT), aspartate aminotransferase (AST), and alkaline phosphatase (ALP) was done using the SPINREACT commercial kits(Ctra Santa Coloma, Spain) as described by manufactures’ manual and guided by the controls using the Urit 3300 machine (Diamond Diagnostics, USA). Case definitions for the various hepatic enzymes were determined based on sex with respect to the reference range considered as up to 40.0 U/L, 38.0 U/L and between 69 and 117.0 U/L for males; and up to 32.0 U/ L, up to 31.0 U/L and between 69 and 117.0 U/L for females; for ALT, AST, and ALP respectively.

Hepatotoxicity was graded using the levels of ALT, AST, and ALP according to classified AIDS Clinical Trials Group classifications as; grade 1 (1.25-2.5^x^ULN), grade 2 (2.51-5.0^x^ULN), Grade 3 (5.1-10^x^ULN ) and, grade 4 (> 10^x^ULN) [11]. Severe hepatotoxicity was defined as Grade 3 or 4. ALT, AST, and ALP grades were discordant. The one with the highest of the three grades was therefore used for classification [9, 12].

### Statistical analysis

The clinical assessment and laboratory results were recorded and double-checked using Microsoft Excel database and analyzed using Statistical Package for the Social Sciences version 20. Categorical variables were expressed as frequencies and proportions and compared using the Chi-square test while continuous variables were expressed as means ± standard error of mean (SEM) and compared to the different treatment durations using the unpaired t-test. The fixed covariates considered to be possible risk factors for the liver elevated enzyme (LEE) at baseline parameters were explored using univariate logistic regression with unadjusted odds ratios and adjusted odds ratios for multivariate analyses to identify risk factors associated with severe hepatotoxicity. The level of significance was set at 5% throughout the analyses.

## Results

### Study population

A total of 100 patients were recruited into the study. Mean (SEM) age of the100 patients with HIV was 36.53(0.56) years and ranged from 18–61 years. The majority 53(53.0%), 43(43.0%), 57(57.0%), and 64(64.0%) were female, within the age range 30–39 years, had CD4^+^ T cell count of < 200 cells/mm^3^ and were placed on TDF + 3TC + EFV treatment respectively (Table 1).

**Table 1:** Socio- demographic and inclusion characteristics of the study population

Characteristics	Factors(Frequency)	%
Age (years) Range	< 30 years (27)	27.0
	30–40 years (43)	43.0
	> 40 years (30 )	30.0
Sex	Female (53)	53.0
	Male (47)	47.0
BMI (kg/m^2^)	Low (47)	47.0
	Normal (31)	31.0
	Overweight (7)	7.0
	Obese (15)	15.0
Level of education	None (5)	5.0
	Primary (51)	51.0
	Secondary (38)	38.0
	Tertiary (6)	6.0
Monthly Income	< 50,000frs (77)	77.0
	> 50,000frs (23)	23.0
Alcohol intake	Yes (56)	56.0
	No (44)	44.0
Cigarette or Tobacco intake	No (73)	73.0
	Yes (27)	27.0
Year of diagnosis	< 1 Year (84)	84.0
	1–3 Years (4)	4.0
	> 3 Years (12)	12.0
CD4 class (cells/ mm^3^)	< 200 (57)	57.0
	> 200 (43)	43.0
WHO stages	stage 1 (14)	14.0
	stage2 (45)	45.0
	stage 3 (37)	37.0
	stage 4 (4)	4.0
Drug type	TDF + 3TC + EFV(64)	64.0
	AZT + 3TC + EFV (22)	22.0
	AZT + 3TC + NVP (14)	14.0

3TC: Lamivudine, AZT: Zidovudine, BMI: Body mass index, EFV: Efavirenz, NVP: Nevirapine; TDF: Tenofovir

### Prevalence of hepatotoxicity

**Fig. 1:**
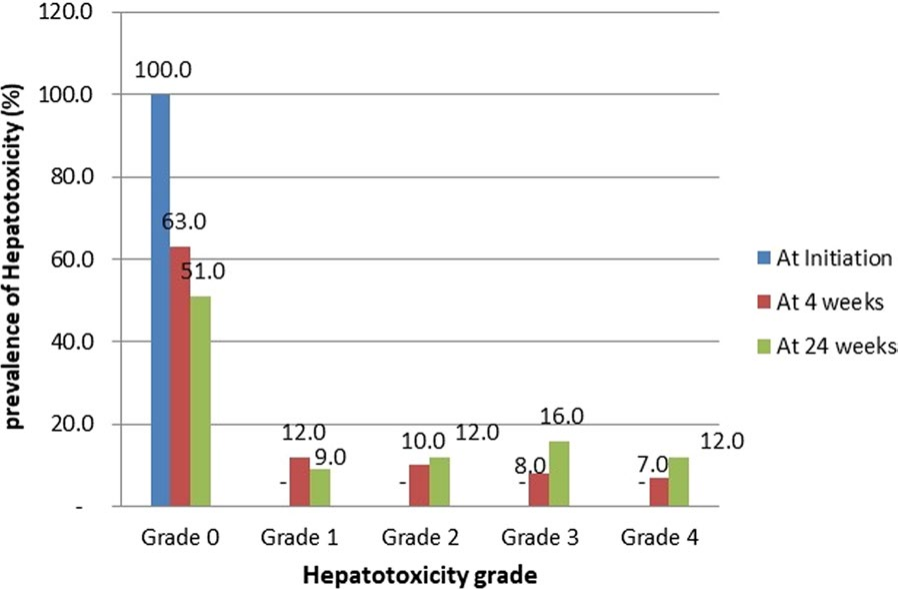
Prevalence (%) of hepatotoxicity grade with respect to duration

All the patients 100 (100%) had no significant LEE at baseline. Using ALT, AST, ALP or a combination of any of them showed that 37(37%) and 49(49%) patients presented with hepatotoxicity at 4 and 24 weeks respectively. This difference was significant (χ^2^ = 68.18; *p* = 0.000). Of this, 22% and 21% had mild-to-moderate (Grades 1 and 2) toxicity at 4 and 24 weeks while 15% and 28% had severe hepatotoxicity (Grades 3 and 4) at 4 and 24 weeks respectively as shown in Fig. 1.

**Fig. 2:**
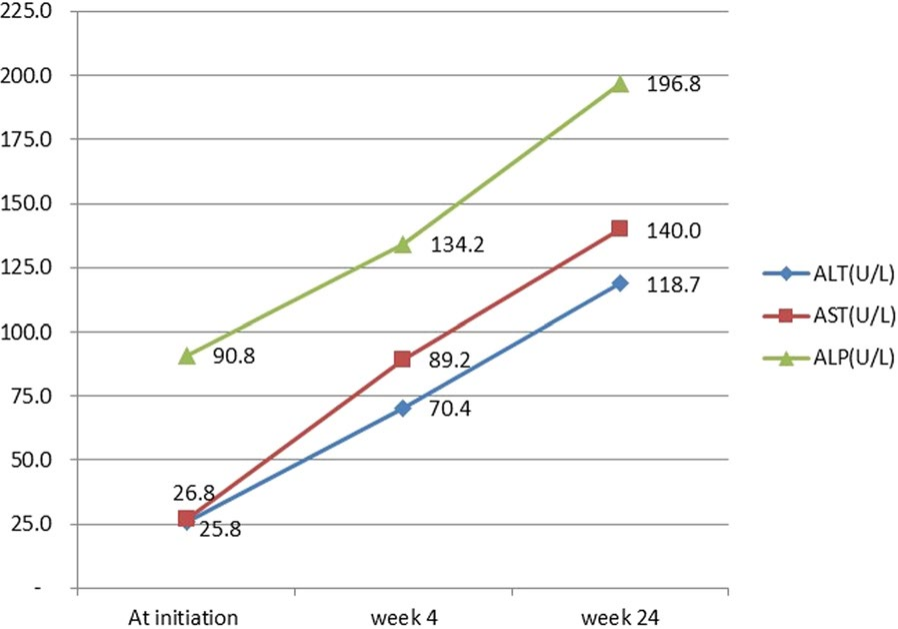
Variation of mean ALT, AST and ALP during the study period

Median ALT, AST and ALP increased significantly with increase in treatment duration at 4weeks and 24weeks; ALT (F = 16.8; *p* = 0.001), AST (F = 11.3;*p* = 0.001) and ALP (F = 7.8; *p* = 0.001) Fig. 2.

#### Risk factors for severe hepatotoxicity at 24 weeks

The mean (SD) BMI and CD4^+^T cell count of patients with severe hepatotoxicity was 27.4 (2.4) kg/m^2^ and 168.0 (25.3) cells/mm^3^ respectively. From univariate regression analyses, severe hepatotoxicity was significantly (p < 0.05) high in the age group < 30 years, males, patients with low monthly income (< 50,000 FRS), low BMI (> 18.5 Kg/m^2^), and patients who took AZT + 3TC + NVP treatment. The prevalence of severe hepatotoxicity among those who consumed alcohol and smoked cigarettes or tobacco only showed a trend (p = 0.07) and (p = 0.09) respectively. In addition level of education, year of diagnosis, WHO staging, were not predictive (P > 0.05) for the development of severe hepatotoxicity (Table 2

**Table 2:** Cox proportional Univariate analysis for Baseline Characteristics of Patients with or without Severe Hepatotoxicity

Characteristics of study the population	Level	Univariate Analysis			
		No N (%)	Yes N (%)	OR(95%CI)	P
Age group (Years)	<30	14 (51.9)	1 3(48.1)	1	0.02
	30–40	33 (76.7)	10 (23.3)	0.15 (0.03–0.65)	
	>40	25 (83.3)	5 (16.7)	0.40 (0.11–1.47)	
Gender	Male	28 (59.6)	19 (40.4)	1	0.04
	Female	44 (83.0)	9 (17.0)	0.26 (0.89–5.28)	
Body Mass Index	Normal	26 (83.9)	5 (16.1)	1	0.02
	Low	29 (61.7)	18 (38.3)	0.38 (0.04–3.35)	
	Overweight	6 (85.7)	1 (14.3)	0.30 (0.11–0.78)	
	Obese	11 (73.3)	4 (26.7)	0.20 (0.02–1.79)	
Level of Education	None	4 (80.0)	1 (20.0)	1	0.89
	Primary	38 (74.5)	13 (25.5)	1.37 (0.14–13.38)	
	Secondary	26 (68.4)	12 (31.6)	1.84 (0.19–18.33)	
	Tertiary	4 (66.7)	2 (33.3)	2 (0.13–31.96)	
Monthly income	< 50,000	51 (66.2)	26 (33.8)	1	0.01
	>50,000	21 (91.3)	2 (8.7)	0.18 (0.04–0.86)	
Alcohol intake	No	33 ( 75)	11 (25 )	1	0.07
	Yes	39 (69.6 )	17 (30.4)	0.40 (0.11–1.47)	
Cigarette or tobacco intake	No	56 (76.7)	17 (23.3)	1	0.09
	Yes	16 (59.3)	11 (40.7)	2.26 (0.88–5.80)	
Year of diagnosis	< 1 Year	61 (72.6)	23 (27.4)	1	0.91
	1–3 Years	3 (75.0)	1 (25.0)	0.88 (0.09–8.94)	
	>3 Years	8 (66.7)	4 (33.3)	1.33 (0.36–4.83)	
CD4 Count values in cells/μl	<200	37 (64.9)	20 (35.1)	1	0.06
	>200	35 (81.4)	8 (18.6)	0.42 (0.161.08)	
WHO stages	Stage 1	8 (57.1)	6 (42.9)	1	0.27
	Stage2	32 (71.1)	13 (28.9)	0.54 (0.16–1.87)	
	Stage 3	30 (81.1)	7 (18.9)	0.31 (0.08–1.19)	
	Stage 4	2 (50.0)	2 (50.0)	1.33 (0.14–12.37)	
Name of ARV regimen	TDF+3TC+EFV	46 (73.0)	18 (27.0)	1	0.01
	AZT+3TC+ EFV	20 (90.9)	2 (9.1)	0.25 (0.05–1.18)	
	AZT+3TC+NVP	6 (42.9)	8 (57.1)	2.86 (0.90–9.04)	

3TC: Lamivudine, AZT: Zidovudine, BMI: Body mass index, EFV: Efavirenz, NVP: Nevirapine; TDF: Tenofovir

Socio-demographic variables (age, gender, BMI, monthly income, alcohol intake, cigarette or tobacco intake, CD4^+^3

**Table 3:** Cox Proportional Multivariate Analysis for Baseline Characteristics of Patients with or without Severe Hepatotoxicity

Characteristics of the study population	Level	Univariate analysis		Multivariate analysis	
		OR (95% CI)	*P*	OR (95% CI)	*P*
Age group (Years)	< 30	1	0.02	1	
	30–40	0.15 (0.03–0.65)		0.16 (0.04–0.72)	0.03
	> 40	0.40 (0.11–1.47)		0.32 (0.08–1.24)	0.1
Gender	Male	1	0.04	1	
	Female	0.26 (0.895.28)		1.78 (0.58–5.49)	0.32
Body Mass Index	Normal	1	0.02	1	
	Low	0.38 (0.04–3.35)		0.33 (0.11-0.99)	0.04
	Overweight	0.30 (0.11-0.78)		0.35 (0.21–4.59)	0.11
Monthly income	< 50,000	1	0.01	1	
	> 50,000	0.18 (0.04–0.86)		0.14 (0.030.79)	0.02
Alcohol intake	No	1	0.07	1	
	Yes	0.40 (0.11-0.47)		0.42 (0.09–1.64)	0.1
Cigarette or tobacco intake	No	1	0.09	1	
	Yes	2.26 (0.885.80)		1.68 (0.51–5.60)	0.39
CD4 Count values	< 200	1	0.06	1	
	> 200	0.42 (0.16–1.08)		0.65 (0.34–2.37)	0.16
Name of ARV regimen	TDF + 3TC + EFV	1	0.01	1	
	AZT + 3TC + EFV	0.25 (0.05–1.18)		0.18 (0.03–0.99)	0.22
	AZT + 3TC + NVP	2.86 (0.90–9.04)		2.44 (0.58–10.26)	0.03

3TC: Lamivudine, AZT: Zidovudine, BMI: Body mass index, EFV: Efavirenz, NVP: Nevirapine; TDF: Tenofovir

## Discussion

With the widespread use of HAART and the availability of new ARV medications, hepatotoxicity has gained prominent attention in the management of patients with HIV/AIDS [3, 9, 13].

Results from this study like in previous studies in other parts of Cameroon and elsewhere have shown a high prevalence of hepatotoxicity among treated patients with HIV/AIDS [6, 9]. HAART-related severe hepatotoxicity results directly either from drug toxicity and/or drug metabolism, hypersensitivity reactions, mitochondrial toxicity, and immune reconstitution inflammatory syndrome [7, 13].

However, in this study, the adverse event did not have a negative impact on the continuation of the treatment. Our data ranged higher than what has been reported from previous studies by Price and Thio (10%) [7], and Sterling et al. (43%) [13]. The observed difference could be associated with differences in the population characteristics, the definition of hepatotoxicity, and the type of HAART being used.

In this study, a decline in the number of cases on Grade 1 and 2 hepatotoxicities with those in Grade 3 and 4, increasing. This change could not be confirmed based of the short period of follow-up. It will therefore be appropriate if a longer period of follow up could be conducted to fully confirm the observed trend on the hepatotoxicity trend [4, 9].

The high prevalence of hepatotoxicity after one month of treatment is a clear indication that some liver diseases such as cytomegalovirus and mycobacterium infections, and AIDS-related neoplasms such as lymphoma and Kaposi’s sarcoma [7] are often associated with HIV infection other than HAART. Of the 38 clients presenting with hepatotoxicity after 4 weeks, 17(44.7%) did not present with hepatotoxicity at 24 weeks. However, it has been reported that the hepatotoxic effects of HAART may resolve with time when the organ gets used to the drug in some patients [14]. Nevertheless, early development of hepatotoxicity can be attributed to mitochondrial toxicity, hypersensitivity reaction, or other opportunistic diseases such as *Mycobacterium* avium complex, *Candidaalbicans, Toxoplasma gondii, Leishmania* species, *Strongyloidesstercoralis*, Cytomegalovirus, etc[7, 10].

On the other hand, since HIV-1 infected patients could be co-infected with other infections, we could not rule out the possibility of these patients having taken othercombinations of non-ART medications orherbal concoctions, which could also have had adverse liver effects either alone or in combination[7]. Secondly, all patients in this study received cotrimoxazole prophylaxis for the treatment of opportunistic infections. All these factors have been reported to cause an increased level of transaminases [4, 5, 7].

Our data reveal that elevated transaminases showed a significant positive linear relationship with an increase in the duration of treatment irrespective of the ARV regimen [5]. Thus, the hepatotoxicity seen in this study was mostly hepatocellular (increases in AST and ALT) than cholestatic (increases in ALP) [13, 14]. The observed severe hepatotoxicity was shown to develop as early as a month after starting treatment [4, 15].

Age was found to be a predictor of severe hepatotoxicity contrary to previous studies, [16]. Our findings showed that severe hepatotoxicity was significantly high among individuals aged < 30 years contrary to previous studies conducted in other regions of Cameroon and elsewhere [5, 9, 13]. The most probable reason for the high prevalence in the group can be attributed to their lifestyle. This group is an active age group in terms of social activities such as high intake of alcohol (63% vs. 53.5% vs. 43.3%) and cigarette smoking (48.1% vs. 30.0% vs. 11.6%)that have been shown to increase the levels of liver enzymes [7, 17, 18]. Secondly, there is a high probability that this group of individuals could also have taken other drugs including herbs to get healed.

Gender was analysed to see if it had an impact on hepatotoxicity. However, in this study, no association was observed according to multivariate analysis. The findings concurred with previous studies [4, 14] but also contrary to other findings elsewhere [8, 13]. Given that the courses of HAART metabolism in humans are not gendered dependent [16], this high prevalence could be associated with other social habits that are common with males rather than females. For instance, more males were found to consume alcohol more (55.4% vs. 44.6%) and cigarettes (62.9% vs. 27.1%) than femalesin this study.

Our observations suggest that body weight could bea major factor in determining the serum level of liver enzymes. Low BMI was found to be an independent predictor of severe hepatotoxicity. A finding that confirms that good nutritional status at the start of HAART could be protective against early hepatotoxicity [3, 4]. Studies have shown that an increase in serum ALT and AST levels could be associated with an increase in BMI as a result of anincrease in dietary fat content [19, 20]. There is a high probability that underweight patients usually consume large amounts of dietary fat. A high level of dietary fat causes higher levels of oxidative stress and lipid peroxidation which results in oxidative stress leading to mitochondrial and DNA damage thus causing hepatocellular injury, activation of hepatic stellate cells hence increased levels of liver enzymes [3, 20]. Furthermore, the use of NVP based regimen as recommended for low BMI patients often causes hepatotoxicity [10].

Monthly income has been shown to play an important role in the life of individuals. In this study low monthly income was another significant predictor of severe hepatotoxicity from the multivariate model. This group of persons usually have increased consumption of dietary fat and thus increase in their BMI that accounted for the increase in the liver enzyme. It has been reported that lower monthly income is associated with negative effects on the quality of life such as anxiety and depression symptoms that worsen their clinical conditions as a result making them prefer herbal and cheaper drug concoctions [21, 22].

The prevalence of severe hepatotoxicity associated with NVP (57.1%) based regimen in this study was similar to other studies [7, 10, 13]. Furthermore, we realise that those who took AZT + 3TC + NVP presented with elevated ALP. This confirms the fact that the use of NVP is associated with cholestatic liver enzyme elevations [13].

Alcohol consumption which is associated with elevated liver enzyme levels is most prevalent in most countries [7, 16, 18]. It has been reported that interactions between alcohol and HAART often appear to be crucial in the development of liver disease especially in HIV-1 infected patients [23].Although the prevalence of severe hepatotoxicity was higher among those patients who took alcohol, alcohol intake only showed a trend in the univariate analysis findings that concur with previous findings [3]. This study findings show that alcohol consumption leads to weight gain hence high-level secretions of the liver enzyme especially in men [24, 25]. There were significantly more men who were overweight and had high BMI with the majority of them having a significant high level of severe hepatotoxicity at 24 weeks. Furthermore, since the AST/ALT ratio at week 24 was < 1.5, it is suggestive that, the experienced liver injury could be associated with HAART [25]. That is why we suggest that interpretation of these findings be taken cautiously these findings on the blood alcohols levels were not interpreted beforehand. In addition, the existing inconsistencies regarding the definitions of alcohol consumption and different types of alcohol products being consumed could have misinterpreted the findings.

Cigarette smoking has significantly been associated with increased levels of ALP and to a lesser extent AST and ALT [17, 26–28]. In this study, however, no significant effect on the liver enzymes was observed contrary to previous studies [26]. The observed high levels of liver enzymes as associated with hepatocellular damage resulting out of the production of numerous toxins like. nicotine that causes lipid peroxidation propagation which in return damages the biological cell membrane of the liver, or the increased production of pro-inflammatory cytokines such as IL-1, IL-6, and TNF-α that results in liver cell injury [28–30].Smoking has also been shown to further aggravate the pathogenic effects on theliver during the processing of alcohol and medications [10, 27, 30]. In addition, smoking could decrease CD4^+^ T cells count and lowering of BMI hence a minor risk to liver toxicity [17, 29].

Interestingly, in our study low CD4^+^T cell count was a minor risk factor for liver toxicity since low CD4^+^T cell count showed a trend with hepatotoxicity only in the univariate analysis. Our data also revealed that the high CD4^+^T cell count after 24 weeks is associated with a higher risk of hepatotoxicity in the adjusted multivariate analysis though it was insignificant. Similar findings have been reported by Price and Thio [7] This result attests to the fact that the use of NVP based regimen and high CD4^+^ T cell count is a risk factor to the development of severe hepatotoxicity [3, 10, 13].

## Conclusions

Age group, Low baseline BMI, low monthly income, and use of AZT + 3TC + NVP regimen were identified as independent risk factors for developing severe hepatotoxicity. Severe hepatotoxicity may arise more frequently with increased use of HAART. Thus, there is a need to educate the community of the potential risk factors (such as nutritional habits, alcohol, and smoking abstention)associated with severe hepatotoxicity in other to improve patient management and care.

### Recommendation

The use of NVP based regimen should not be recommended for low BMI patientsand patients > 40 years. The HAART regimen of those with severe hepatotoxicity should first be altered and followed up before discontinuation.

### Limitations

This study had some limitations as follows. The study design did not allow us to establish age, gender, and type of HAART balance ratio at recruitment. In addition, adequate drug adherence assessment and serum drug level estimation could not be performed due to the lack of facilities for such tests. The use of traditional herbs was not considered in this study with concurrent non TB medication. Lastly, liver histology could not be performed in these patients.

## Data Availability

The dataset used for analyzed in this study is available from the corresponding author on request.

## Abbreviations

3TC: Lamivudine
ALP: Alkaline phosphatase
ALT: Alanine aminotransferase
AST: Aspartate aminotransferase
AZT: Zidovudine
BMI: Body mass index
EFV: Efavirenz
NVP: Nevirapine
SEM: Standard error of mean
TDF: Tenofovir
